# A Bidirectional Wavelength Division Multiplexed (WDM) Free Space Optical Communication (FSO) System for Deployment in Data Center Networks (DCNs)

**DOI:** 10.3390/s22249703

**Published:** 2022-12-11

**Authors:** Fady El-Nahal, Tianhua Xu, Dokhyl AlQahtani, Mark Leeson

**Affiliations:** 1 School of Engineering, University of Warwick, Coventry CV4 7AL, UK; 2 Department of Electrical Engineering, Islamic University of Gaza, Gaza P.O. Box 108, Palestine; 3 School of Electrical and Computer Engineering, Georgia Institute of Technology, Atlanta, GA 30332-0250, USA; 4 Department of Electrical Engineering, Prince Sattam bin Abdulaziz University, Al-Kharj 16278, Saudi Arabia

**Keywords:** free space optics, wavelength division multiplexed, wireless data center networks

## Abstract

Data centers are crucial to the growth of cloud computing. Next-generation data center networks (DCNs) will rely heavily on optical technology. Here, we have investigated a bidirectional wavelength-division-multiplexed (WDM) free space optical communication (FSO) system for deployment in optical wireless DCNs. The system was evaluated for symmetric 10 Gbps 16—quadrature amplitude modulation (16-QAM) intensity-modulated orthogonal frequency-division multiplexing (OFDM) downstream signals and 10 Gbps on-off keying (OOK) upstream signals, respectively. The transmission of optical signals over an FSO link is demonstrated using a gamma–gamma channel model. According to the bit error rate (BER) results obtained for each WDM signal, the bidirectional WDM-FSO transmission could achieve 320 Gbps over 1000 m free space transmission length. The results show that the proposed FSO topology offers an excellent alternative to fiber-based optical interconnects in DCNs, allowing for high data rate bidirectional transmission.

## 1. Introduction

The exponential increase in Internet traffic has thrust data centers into the era of Zettabytes. The bulk of this increased traffic is due to videos and related machine-learning applications [1]. In the cloud computing world of today, access to enormous volumes of data, computation, and storage resources is more crucial than ever before. Mega-data centers with hundreds of thousands of servers that are networked by intra-data center inter-connects that typically extend from 300 m (known as short-reach) to more than 2 km (known as long-reach) using single mode fiber (SMF) have become increasingly prevalent. GaAs-based vertical cavity surface emitting lasers (VCSELs) that operate in the 850 nm window are used in the transceivers for short-reach intra-data center interconnects [2]. The data are modulated using 4-level pulse amplitude modulation (PAM-4) before being sent over MMF. Furthermore, wavelengths of 1310 or 1550 nm wavelengths are used with Silicon photonics or InP transceivers, respectively, for long-reach inter-data center interconnects [3,4].

Data center networks (DCNs) are vital to interconnect a multitude of servers with high-speed, low-latency links, resulting in substantial expense and complexity associated with wire deployment, maintenance, and upgrades [1,5]. Wireless data center networks (WDCNs) can provide better throughput and efficient bandwidth utilization. Moreover, they eliminate the need for expensive and power-hungry switches and reduce the cost and complexity of the cabling. High-speed wireless networks can be created using cutting-edge technologies such as millimeter wave and free-space optical (FSO) communications [6,7,8]. High-capacity FSO connections can be achieved by employing wavelength division multiplexing (WDM) technology that provides a high fan-out (i.e., a significant number of channels per FSO connection) and elastic topologies [9]. Indoor FSO links can achieve superior performance at longer distances due to the customary conditions of DCNs. They are less subject to optical channel impairments such scintillation, alignment problems, and atmospheric turbulence [5].

Recently, FSO-based systems have been investigated for deployment as access networks, both for fronthaul and backhaul [10]. Such systems have several advantages, including high bandwidth, spectrum utilization without a license, reduced power consumption, smart hardware, as well as immunity against electromagnetic interference (EMI). Moreover, FSO systems are less expensive to deploy than optical fiber-based communication systems. There are significant set-up and maintenance costs and time associated with optical access networks, especially passive optical networks (PONs) and radio over fiber (RoF) systems. Therefore, FSO communication systems are expected to significantly reduce both deployment cost and time [4,5].

FSO is mainly impacted by atmospheric attenuation and random variation of signal intensity and phase caused by atmospheric turbulence, known as intensity scintillation [11]. This phenomenon is comparable to multipath fading in radio frequency (RF) wireless communication and is caused by random fluctuations in the refractive index of the atmosphere resulting from variations in temperature and pressure [12]. Various FSO statistical channel models are used for turbulence estimation, such as the log-normal model, K model, negative exponential model, gamma–gamma model, and log-normal Rician model [13]. The gamma–gamma FSO model is commonly used to simulate atmospheric turbulence and attenuation, with characteristics ranging from mild to strong [4].

FSO systems have been extensively investigated in recent years. The performance of an FSO link based on on–off keying (OOK) coding has been investigated and improved by employing a coherent detection and dynamic decision threshold in [14]. An efficient blind detection of OOK signals utilizing an instantaneous channel fading coefficient is proposed in [15]. A visible light FSO link employing a hybrid intensity modulation and direct detection (IM/DD) system is proposed in [16]. The spectral efficiency of the system has been enhanced by integrating the asymmetrically clipped optical orthogonal frequency division multiplexing (OFDM) and OOK modulation formats. An FSO link employing hybrid modulation formats such as quadrature amplitude and multi-pulse position modulation techniques has been studied with and without intensity turbulence [17]. Padhy and Patnaik [18] investigated the performance of an FSO link based on a differential phase shift keying (DPSK) modulation format with and without incorporating Manchester coding. A WDM-FSO system integrated with a fiber access network for wireless traffic was experimentally investigated in [19]. FSO systems were integrated with PONs in [20], where 10 Gbps OOK data were transmitted. A full-duplex architecture for the transmission of ultrawideband (UWB) signals over an FSO network that offers the benefits of installation simplicity and mobility was presented in [21]. All-optical generation and transmission of multiple ultrawideband signals to radio access units over an FSO link was demonstrated in [11] using a centralized frequency comb source. The performance of an all-optical relay-assisted FSO system was investigated in [22] for OOK and DPSK modulation formats. In [23], a single lightwave source was used for delivering baseband, FSO, and MMW traffic simultaneously in a PON architecture. A Hybrid PON-FSO strategy was introduced in [24] to create a more cost-effective solution for fronthaul networks in congested metropolitan areas compared to solo PON systems, primarily owing to the FSO technology. The main optical wireless communication (OWC) technologies, algorithms, concepts, and configurations that could improve the performance of the next generation of data centers are discussed in [25]. Recent developments in FSO DCN design have been discussed in [26], as well as current designs that utilize cellular technology to realize point-to-point Line-of-Sight (LOS) links. An OWC DCN architecture based on a diffractive grating and a fast tunable transmitter was demonstrated in [27], where different modulation formats (NRZ-OOK and PAM4), as well as different sizes of DCN, have been evaluated.

This paper reviews recent and prospective DCN technologies to meet aggregate capacity demands. Moreover, we propose and demonstrate a novel bidirectional WDM-FSO system based on IM/DD OFDM for employment in OWC-DCNs. In order to increase capacity and reduce costs, a wavelength scheme based on reflective semiconductor optical amplifiers (RSOAs) has been implemented, which requires no optical source for upstream transmissions. The findings here are comparable to those found in the literature, such as [19,23,24]. In contrast to the basic modulation formats used in the literature, we have employed a novel technique based on OFDM technology and wavelength reuse. Here, 10 Gbps OFDM and OOK FSO signals can be transmitted over a 1000 m free space link for downstream and upstream traffic, respectively. The proposed WDM FSO system offers cost-effective transmission of high-data-rate signals, as well as mobility and a shortened deployment time.

## 2. Overview of Data Center Networks (DCNs)

There is a growing demand for DCNs due to the increasing data traffic. Researchers have been working to improve architecture at all levels to enhance performance. Lately, Facebook, Google, and Microsoft have begun to use multiple wavelength links through coarse wavelength division multiplexing (CWDM) [28,29,30]. There are several structures that have been proposed for enhancing DCN performance using high bandwidth density optics, as well as optical switches [31,32]. DCNs have developed rapidly in recent years. Large-scale data centers migrated to optical transmission technologies when they upgraded the 1 Gbps link data rate to 10 Gbps by the end of 2010. Google introduced optical links in its data centers in 2007 using 10 Gbps VCSELs and multimode fiber-based small form-factor pluggable (SFP) transceivers for distances up to 200 m. The data rates have risen from 10 to 40 Gbps, 40 to 100 Gbps, and even higher up to 400 Gbps. Lately, 100 Gbps links have been employed in production data centers. Moreover, 400 Gbps transceivers have recently been standardized for short-range (0.5–10 km) intra-data-center interconnects over standard SMF [33,34].

Optical transceivers are the core of DCNs and consist of a laser, modulator, multiplexer/demultiplexer (Mux/DeMux), and photodetector. Lasers and modulators can be paired, for instance, in directly modulated VCSELs. Transceivers based on VCSELs and parallel fibers are currently the most prevalent technology in DCNs. As the traffic increases, bandwidth needs are increasing dramatically. Therefore, WDM technology is essential for ultra-wide bandwidth, low-energy links. In a WDM system, several wavelength signals are combined using (de)multiplexers and transmitted over fiber. A distributed feedback laser array is usually used with Mach–Zehnder modulators (MZMs) or electro-absorption modulators (EAMs). To achieve ultra-high data rates, however, several laser sources are needed. Therefore, comb lasers emitting over several individual wavelengths are an attractive option. A multi-wavelength comb source architecture could include DeMux/Mux stages as well as broadband modulators. In this architecture, the ultra-wideband signal is spectrally divided into different sub-bands for separate processing. At the source, the ultra-wideband signal is created by integrating a large number of optical sub-bands, each modulated by an input RF signal. An ultra-wideband communication channel can be built by tightly multiplexing a very large array of sub-bands for a variety of purposes, including optical interconnections [1].

Optical communication networks are increasingly in need of higher bandwidth due to the ever-increasing demand. Consequently, there is an urgent need to improve modulation speeds. Conventional modulation schemes for fiber optic networks typically utilize dense wavelength division multiplexing (DWDM) of separately modulated OOK signals. Nonetheless, the limited total optical spectrum and the low bit rates achievable per optical channel limit the total bandwidth available. In recent years, wideband modulation schemes using higher spectral efficiency modulation and multi-tone carriers such as optical OFDM have gained attention [35,36,37]. OFDM is a multicarrier high data rate transmission technique used in communication systems. To transmit the overall high data rate, the OFDM system employs several lower-rate orthogonal subcarriers.

## 3. WDM-FSO Architecture

Acronyms introduced in the manuscript and their definitions are listed in Table 1.

**Figure 1:**
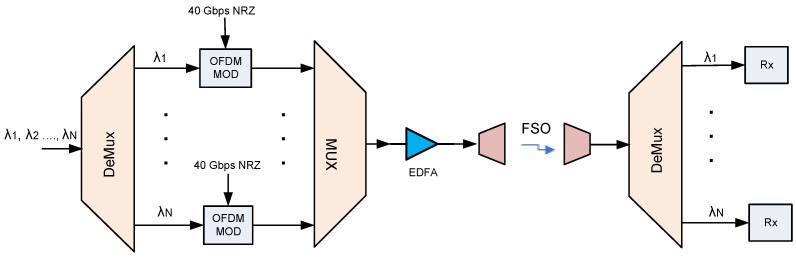
The WDM FSO architecture.

Figure 1 shows a WDM-FSO architecture based on a multi-wavelength comb source, including DeMux and Mux stages, as well as broadband modulators. At the source, the UWB is created by integrating several optical wavelengths, each modulated by an input RF OFDM signal. An OFDM signal is produced as a sum of orthogonal subcarriers, which are modulated using different encoding techniques, for instance, quadrature amplitude modulation (QAM). For this work, the system was designed using 16-QAM modulation, which offers higher transmission rates and better spectral efficiency. Additionally, FSO systems benefit from the use of OFDM due to their resilience to multipath fading, frequency selective fading, and intersymbol interference (ISI), thereby increasing their transmission range. The information data are processed by a QAM sequence encoder to transform the data bits into symbols (4 bits per symbol for 16-QAM). The QAM symbols are then modulated into multiple orthogonal sub-carriers and the QAM-OFDM signal generated is used to modulate the optical WDM signal. A non-return to zero (NRZ) $2^{23}-1$ pseudo random bit stream (PRBS) sequence with a data rate of 40 Gbps has been generated for each WDM channel. In the OFDM modulator, the number of OFDM subcarriers is 512 and the number of Fast Fourier Transform (FFT) points is 2048, so the generated bit rate is (512/2048) ×40=10 Gbps. ISI between OFDM symbols is avoided by adding a cyclic prefix of 100 for each OFDM symbol after the Inverse FFT (IFFT). The proposed system has an N=32 wavelengths WDM FSO link (32×10 Gbps) that covers the frequency range of 193.1 to 194.65 THz (1552.52 to 1540.16 nm) and has a total transmission capacity of 320 Gbps. Cosine roll-off filters are employed to shape pulses before transmission to minimize ISI. A laser source and an MZM are used to up-convert the OFDM-modulated signal from the electrical to the optical domain. Then the resulting optical signal is multiplexed with other WDM signals, amplified using a gain-flattened Erbium-doped fiber amplifier (EDFA), and sent over the FSO channel.

Next, the WDM signals are separated by a DeMux, where various wavelength signals are directed to the desired receiver. At the receiver, each WDM signal is converted to an electrical signal by a PIN photodetector (PD). An electrical amplifier then amplifies the received electrical signal. The amplified signal is then downconverted and recovered using a quadrature demodulator. After that, using an OFDM demodulator, the transmitted QAM symbols are successfully retrieved. Then the received symbols are converted into bits by a QAM sequence decoder based on the number of bits per symbol.

A bidirectional WDM FSO system can be achieved by replicating the original topology for upstream transmission. However, a wavelength reuse scheme can be employed to reduce the cost since there is no optical source needed for upstream transmission. These systems maximize wavelength utilization by using the same wavelength for upstream and downstream channels. Downstream wavelength signals are remodulated with upstream data and then sent back as upstream signals [38,39]. The wavelength reuse scheme has been widely employed in WDM-PONs based on the RSOA, which can be used as an uplink colorless transmitter and modulator, thanks to its properties of optical gain, integration capability, and wide optical bandwidth [40].

**Figure 2:**
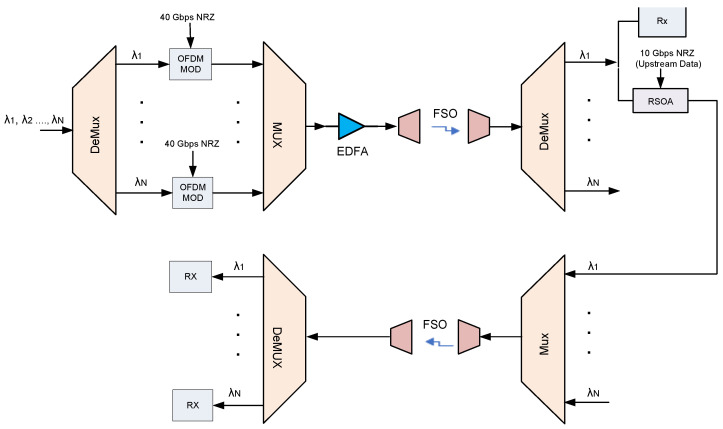
The bidirectional WDM FSO architecture.

Figure 2 depicts a bidirectional FSO WDM system based on a wavelength reuse scheme. It is similar to the original topology shown in Figure 1. The difference is that the demultiplexed WDM signals at the receiver side are passed through 3 dB optical splitters, where half of the WDM-OFDM modulated signal is fed to an OFDM receiver. For up-link, the other half of the downstream modulated signal is directly remodulated by the RSOA to generate the 10 Gbps NRZ OOK PRBS $2^{23}-1$ upstream signal. As the seeding power of an RSOA has a significant effect on its modulation characteristics, a variable optical attenuator (VOA) is used to keep the power at the input of RSOA at a level in which it operates in the gain–saturation regime. The OOK signals generated are combined using a Mux and then launched back via an FSO channel to form upstream data, where they are separated by a DeMux, and each OOK signal is directly detected by a PIN receiver.

## 4. FSO Channel Model

There are a variety of factors that degrade optical signals transmitted over FSO links; primarily, the attenuation of the laser power in the atmosphere and the geometrical loss caused by the spreading of the transmitted beam between the transmitter and the receiver.

The link equation can be expressed as [24]:

(1) $$P_{Received}=P_{Transmitted}\frac{d_{R}^{2}}{{\left(d_{T}+\theta R\right)}^{2}}10^{-\alpha \frac{R}{10}},$$

where $d_{R}$ is the receiver aperture diameter (m), $d_{T}$ is the transmitter aperture diameter (m), θ is the beam divergence (milliradians), *R* is the range (km) and α is the atmospheric attenuation (dB km^−1^).

Additional losses include the transmitter and receiver losses caused by the fiber–telescope interface, mis-pointing and coupling deficiencies. Furthermore, there is a significant loss due to scintillation. Intensity scintillation results mainly from the random variations in temperature and pressure of the air, causing index-of-refraction fluctuations, or in other words, optical turbulence. There are various channel models that can be used to describe the intensity scintillation of an optical signal. The gamma–gamma channel model can mimic a broad range of turbulence conditions.

In this case, the probability density function of the normalized light intensity *I* is given by:

(2) $$P\left(I\right)=\frac{2{\left(\alpha \beta \right)}^{\frac{\alpha +\beta }{2}}}{\Gamma (\alpha )\Gamma (\beta )}I^{\frac{\alpha +\beta }{2}-1}K_{\alpha -\beta }\left(2\sqrt{\alpha \beta I}\right)$$

(3) $$\alpha =exp\left[\frac{0.49\sigma_{R}^{2}}{{\left(1+1.11\sigma_{R}^{\frac{12}{5}}\right)}^{\frac{5}{6}}}\right]-1$$

(4) $$\beta =exp\left[\frac{0.51\sigma_{R}^{2}}{{\left(1+0.69\sigma_{R}^{\frac{12}{5}}\right)}^{\frac{5}{6}}}\right]-1$$

α and β are the numbers of small and large turbulence cells, Γ(·) is the gamma function and $K_{\alpha -\beta }\left(\cdot \right)$ is a modified Bessel function of the second kind [13].

The intensity variance $\sigma_{R}^{2}$, which directly depends on the magnitude of atmospheric turbulence, is given by:

(5) $$\sigma_{R}^{2}=1.23C_{n}^{2}k^{\frac{7}{6}}z^{\frac{11}{6}}$$

where $C_{n}^{2}$ is the refractive index structure parameter with value ranges from $10^{-13}$$m^{\frac{-2}{3}}$ for strong turbulence to $10^{-17}\;m^{\frac{-2}{3}}$ for weak turbulence, $k=\frac{2\pi }{\lambda }$ is the optical wavenumber, and *z* is the range of the FSO link. Channel time fluctuations are calculated based on the theoretical quasi-static model, also known as the frozen channel model. This model is based on the idea that channel fading stays the same over the course of a frame of symbols (coherence time), changing to a new value between frames.

## 5. Results and Discussion

**Figure 3:**
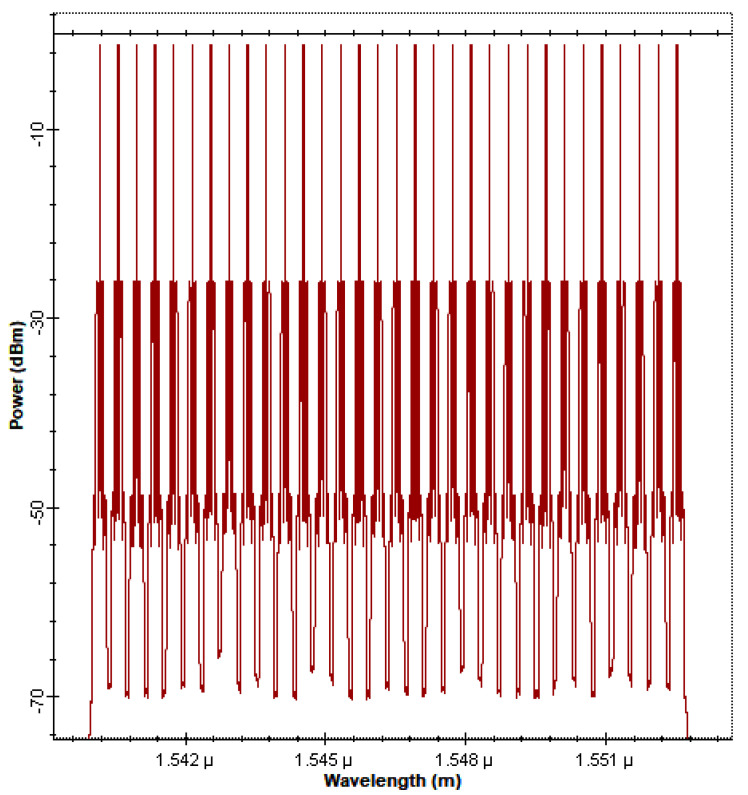
Spectrum of proposed 32-channel WDM-FSO system.

The WDM-FSO architectures in Figure 1 and Figure 2 were designed and analyzed using the Optiwave OptiSystem simulation tool for optical system design [41]. The parameters used in the system simulation are listed in Table 2. Figure 3 depicts the spectrum of the proposed FSO system after the gain flattening EDFA stage of amplification. It is clear that the proposed 32—channel FSO link has achieved gain flatness and that the power levels on all channels have been stabilized.

**Figure 4:**
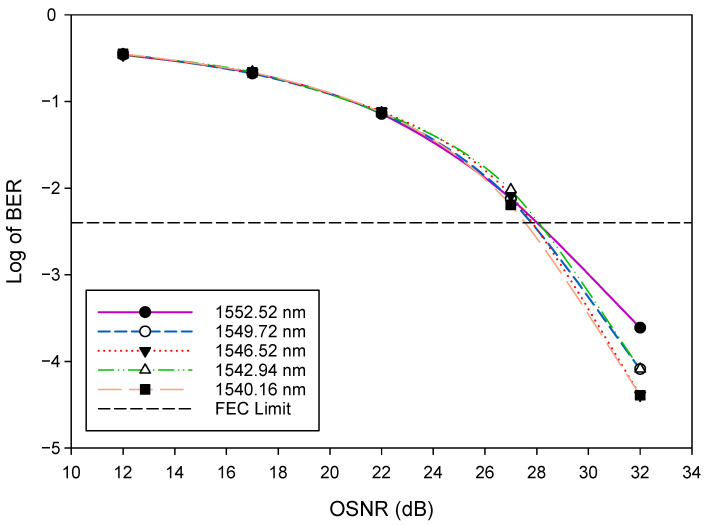
BER versus OSNR plots for selected WDM signals.

**Figure 5:**
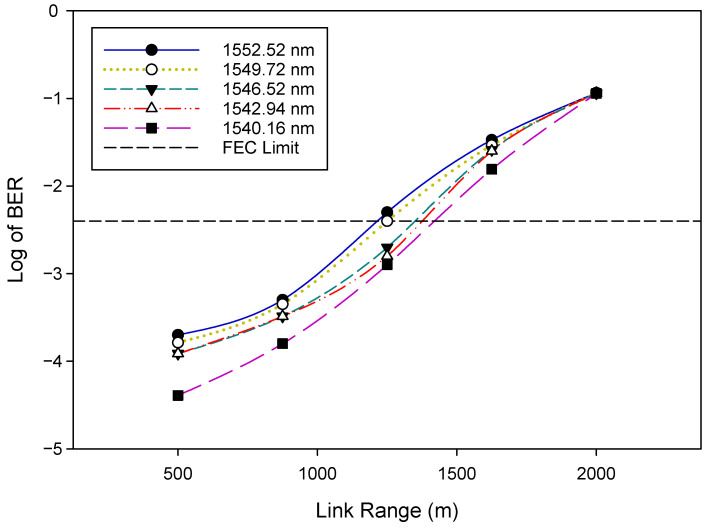
BER versus Link range plots for selected WDM signals.

Firstly, we evaluated the general WDM-FSO architecture shown in Figure 1. The performance of the FSO signal was investigated by measuring the bit error rate (BER) versus the optical signal-to-noise ratio (OSNR). The BER results after 500 m FSO transmission for five selected WDM signals: 1552.52 nm ($\lambda_{1}$), 1549.72 nm ($\lambda_{8}$), 1546.52 nm ($\lambda_{16}$), 1542.94 nm ($\lambda_{24}$), and 1540.16 nm ($\lambda_{32}$) are shown in Figure 4. The results indicate that, as would be expected, the BER improves as the OSNR increases. In addition, the BER deteriorates gradually when the FSO signal changes to a longer wavelength. For instance, at OSNR = 32 dB, the BER rises from $2.5\times 10^{-4}$ for $\lambda_{1}$ to $4.1\times 10^{-5}$ for $\lambda_{32}$. We also indicate in Figure 4 the forward error correction (FEC) limit [42], which represents the maximum BER level at which FEC could be used to bring the FEC to an acceptable level (∼$10^{-9}$ for the level shown). The OSNR of the WDM signals changes slightly from 28 dB for $\lambda_{1}$ to 27.5 dB for $\lambda_{32}$, respectively, at this FEC limit of ∼$3.8\times 10^{-3}\left(-2.4\right)$. Figure 5 depicts the BER performance of the FSO communication system for various link ranges. Again, in line with expectations, the BER performance of the system deteriorates as the transmission distance increases. It is clear from the results in Figure 4 and Figure 5 that when the FSO signal moved to the longer wavelength position gradually, the BER performance slowly degraded. At the FEC limit, for instance, 1552.52 nm ($\lambda_{1}$) could reach ≈1200 m, whereas 1540.16 nm ($\lambda_{32}$) could reach ≈1400 m, respectively. Consequently, the aggregate FSO signal was capable of 1200 m free space transmission.

**Figure 6:**
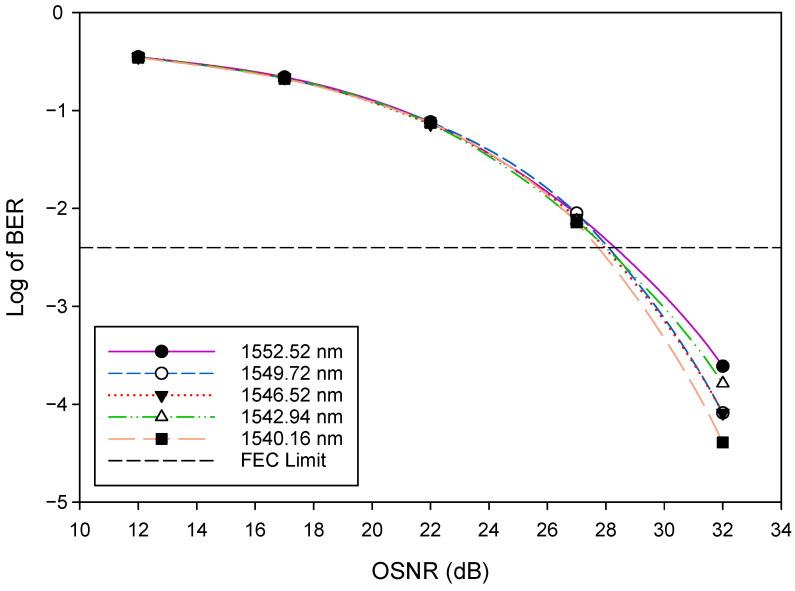
BER versus OSNR plots for downlink.

**Figure 7:**
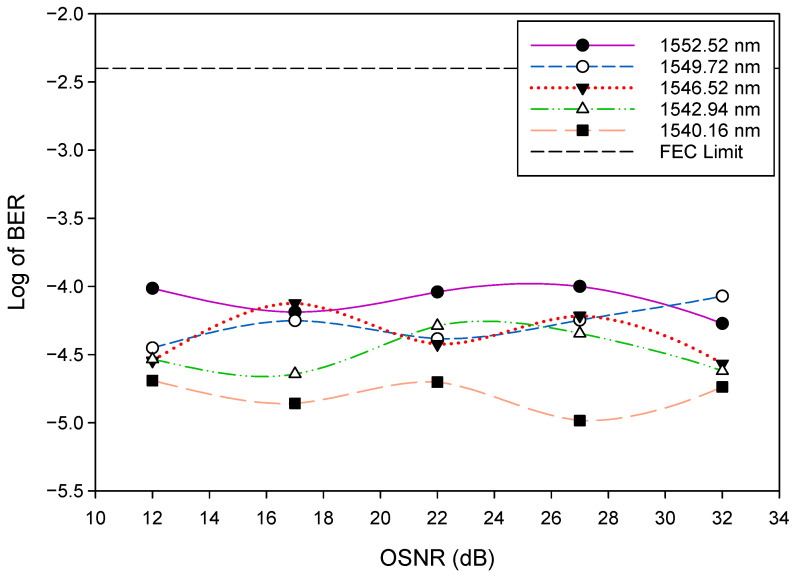
BER versus OSNR plots for uplink.

Secondly, we have evaluated the performance of the bidirectional WDM-FSO architecture in Figure 2. Figure 6 and Figure 7 show the monitored BER performance of the OFDM downstream and OOK upstream FSO traffic, respectively, using wavelengths 1552.52 nm ($\lambda_{1}$), 1549.72 nm ($\lambda_{8}$), 1546.52 nm ($\lambda_{16}$), 1542.94 nm ($\lambda_{24}$), and 1540.16 nm ($\lambda_{32}$) over a 500 m free space transmission length. The downlink results in Figure 6 are comparable to those of the original topology in Figure 4, with BER clearly improving with OSNR. Additionally, when the FSO signal gradually shifts to a longer wavelength, the BER performance degrades steadily. For example, at OSNR =32 dB, the BER increases from $2.5\times 10^{-4}$ for $\lambda_{1}$ (1552.52 nm) to $4\times 10^{-5}$ for $\lambda_{32}$ (1540.16 nm). Moreover, at the FEC limit, the OSNR of the WDM signals varies marginally from 28.3 dB for wavelength $\lambda_{1}$ to 27.6 dB for wavelength $\lambda_{32}$.

**Figure 8:**
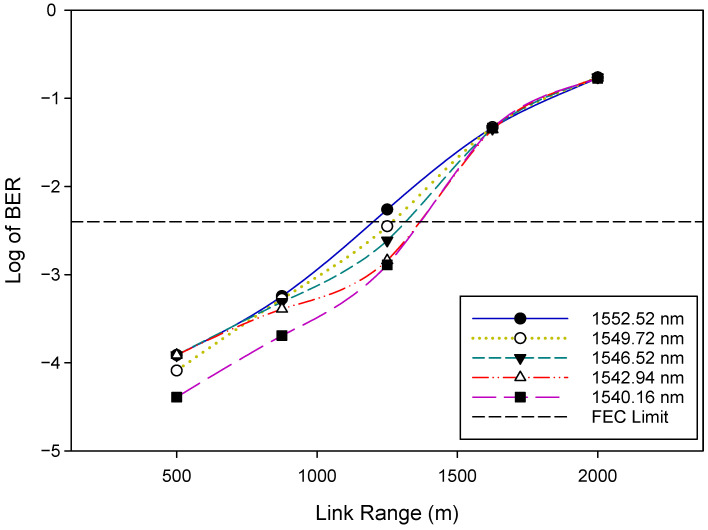
BER versus Link range plots for downlink.

**Figure 9:**
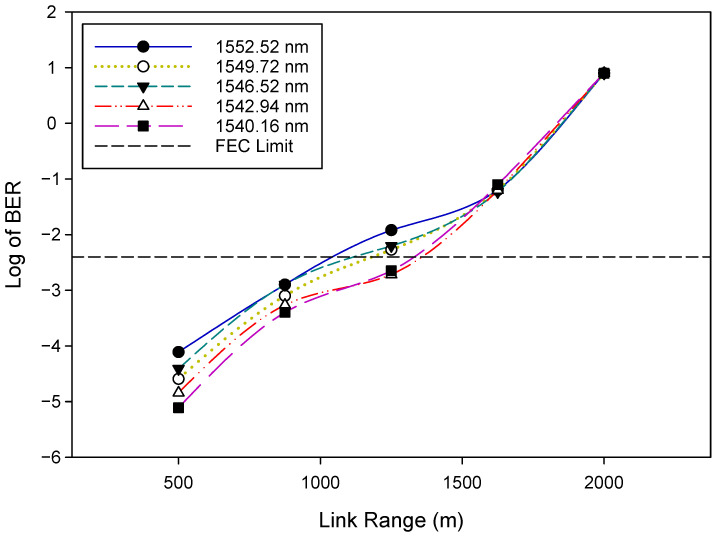
BER versus Link range plots for uplink.

However, for the uplink, it is plain from Figure 7 that there is a slight variation in BER with the increase in OSNR for each WDM signal, which is expected as the RSOA is operating in the gain saturation region. Furthermore, it is apparent that the BER performance worsens as the FSO signal gradually changes to a longer wavelength (from $\lambda_{32}=1540.16$ nm to $\lambda_{1}=1552.52$ nm). The parameters of the RSOA used here are listed in Table 3. Figure 8 and Figure 9 illustrate the BER performance of the downstream and upstream FSO traffic, respectively, over a variety of link ranges. The system’s BER performance degrades as transmission distance increases, as shown in Figure 8 and Figure 9. Moreover, we notice that as the FSO traffic advances to longer wavelengths gradually, the corresponding BER performance becomes relatively poor, as seen in the results obtained. Therefore, shorter wavelength FSO traffic can achieve better BER performance and a longer range, as illustrated in Figure 8 and Figure 9. At the FEC limit, for instance, 1552.52 nm ($\lambda_{1}$) could reach ≈1200 m downstream and ≈1000 m upstream, respectively, whereas 1540.16 nm ($\lambda_{32}$) could reach ≈1400 m downstream and ≈1300 m upstream, respectively. Consequently, the aggregate FSO signal was capable of 1000 m bidirectional FSO transmission. The proposed WDM-FSO network had no visible crosstalk since the downstream, and the upstream signals traveled on different paths across the FSO link. The results illustrate that downstream signals can achieve a longer transmission distance than upstream signals due to the improved link performance that results from the use of OFDM formats. Additionally, the results demonstrate that the upstream signals are relatively unaffected by the Rayleigh backscattering noise caused by the downstream signals. This has been achieved by operating the RSOA in the gain saturation region to remove the downlink modulation on the seeding wavelength. Moreover, the results indicate that shorter wavelength FSO traffic can achieve better BER performance and an extended range than longer wavelengths. This is consistent with the experimental results obtained in [19] and can be explained by the fact that at longer wavelengths, the attenuation resulting from atmospheric absorption or scattering increases slightly, degrading the performance of the system.

## 6. Conclusions

A review of current and prospective DCN technologies to accommodate the aggregate capacity demands has been provided. We demonstrated a 320 Gbps (32×10 Gbps) bidirectional WDM-FSO access system for use in DCNs, where data speeds are typically high and air turbulence is minimal. The wavelength reuse scheme based on wavelength-seeded RSOAs has been employed to decrease the cost of WDM-FSO topologies. The WDM-FSO scheme was implemented with a centralized multi-wavelength comb source and direct detection. An OFDM-modulated signal has been transmitted over the downlink. For the uplink, the downstream OFDM signal was re-modulated as an OOK signal by an RSOA at the receiver. BER measurements of FSO signals show that the proposed WDM-FSO system may achieve bidirectional 10 Gbps of traffic for each wavelength over a 1000 m free space transmission length. The BER results show that the proposed high data rate FSO topology offers an excellent alternative to fiber-based optical interconnects in DCNs or point-to-point links in PONs, allowing for high data rate bidirectional transmission.

## Figures and Tables

**Table 1:** List of acronyms.

Acronym	Description
WDM	wavelength division multiplexing
FSO	free space optical communication
DCNs	data center networks
16-QAM	16-quadrature amplitude modulation
VCSEL	vertical cavity surface emitting laser
PAM-4	4-level pulse amplitude modulation
OFDM	orthogonal frequency division multiplexing
EDFA	erbium-doped fiber amplifier
RSOA	reflective semiconductor optical amplifier
PONs	passive optical networks
OWC	optical wireless communication
RoF	radio over fiber

**Table 2:** Simulation parameters.

Parameter	Value
Laser power	10 dBm
Length of FSO link	500 m
FSO attenuation	3 dB km^−1^
Beam divergence (θ)	2 mrad
Transmitter aperture diameter ($d_{T}$)	5 cm
Receiver aperture diameter ($d_{R}$)	20 cm
No. of WDM channels	32
Spacing between WDM channels	50 GHz
Responsivity of PIN	1 A W^−1^
PIN thermal power density	$15\times 10^{-24}$ ${\mathrm{WHz}}^{-1}$
PIN dark current	10 nA
Gain of electrical amplifier	10 dB
Electrical amplifier power spectral density	−60 dBm Hz^−1^

**Table 3:** Main RSOA parameters.

Parameter	Value
Input Facet Reflectivity	$50\times 10^{-6}$
Output Facet Reflectivity	$50\times 10^{-6}$
Active Length	0.0006 m
Taper Length	0.0001 m
Width	$0.4\times 10^{-6}$ m
Height	$0.4\times 10^{-6}$ m
Optical Confinement Factor	0.45
Nonlinear Gain Parameter	$112\times 10^{-6}$ $m^{3}$

## Data Availability

Not applicable.
